# Detection of AR-V7 in Liquid Biopsies of Castrate Resistant Prostate Cancer Patients: A Comparison of AR-V7 Analysis in Circulating Tumor Cells, Circulating Tumor RNA and Exosomes

**DOI:** 10.3390/cells8070688

**Published:** 2019-07-08

**Authors:** Mohammed Nimir, Yafeng Ma, Sarah A. Jeffreys, Thomas Opperman, Francis Young, Tanzila Khan, Pei Ding, Wei Chua, Bavanthi Balakrishnar, Adam Cooper, Paul De Souza, Therese M. Becker

**Affiliations:** 1 Centre for Circulating Tumour Cell Diagnostics and Research, Ingham Institute for Applied Medical Research, 1 Campbell St, Liverpool, NSW 2170, Australia; 2 South Western Clinical School, University of New South Wales, Goulburn St, Liverpool, NSW 2170, Australia; 3 School of Medicine, Western Sydney University, Campbelltown, NSW 2560, Australia; 4 Liverpool Hospital, Elizabeth St & Goulburn St, Liverpool, NSW 2170, Australia; 5 School of Medicine, University of Wollongong, Wollongong, NSW 2522, Australia

**Keywords:** prostate cancer, CTC, AR, AR-V7, ctRNA, exosome

## Abstract

Detection of androgen receptor (AR) variant 7 (AR-V7) is emerging as a clinically important biomarker in castrate resistant prostate cancer (CRPC). Detection is possible from tumor tissue, which is often inaccessible in the advanced disease setting. With recent progress in detecting AR-V7 in circulating tumor cells (CTCs), circulating tumor RNA (ctRNA) and exosomes from prostate cancer patients, liquid biopsies have emerged as an alternative to tumor biopsy. Therefore, it is important to clarify whether these approaches differ in sensitivity in order to achieve the best possible biomarker characterization for the patient. In this study, blood samples from 44 prostate cancer patients were processed for CTCs and ctRNA with subsequent AR-V7 testing, while exosomal RNA was isolated from 16 samples and tested. Detection of AR and AR-V7 was performed using a highly sensitive droplet digital PCR-based assay. AR and AR-V7 RNA were detectable in CTCs, ctRNA and exosome samples. AR-V7 detection from CTCs showed higher sensitivity and has proven specificity compared to detection from ctRNA and exosomes. Considering that CTCs are almost always present in the advanced prostate cancer setting, CTC samples should be considered the liquid biopsy of choice for the detection of this clinically important biomarker.

## 1. Introduction

Advanced prostate cancer (PC) tends to be initially hormone sensitive and is treated with androgen deprivation therapy (ADT). However, resistance to first line therapy usually develops in approximately 20–40% of patients, referred to as castrate resistant prostate cancer (CRPC) [1]. Most commonly, PC cells become resistant through molecular changes of the androgen receptor (AR), such as mutations, gene amplification, and, more recently reported, the expression of transcript AR variants [2]. In particular, the expression of AR variant 7 (AR-V7), the most abundant and clinically relevant of all variants, has been implicated as a cause of CRPC [3,4]. The translated AR-V7 protein is truncated and lacks the ligand binding domain as well as sequences important for stability and maybe cellular localization [5,6]. Importantly, intracellular AR-V7 predominantly localizes to the nucleus and displays ligand independent transcriptional activity, which is thought to be fundamental in its ability to promote ADT resistance [3,6,7].

Due to its role in ADT resistance several therapies, such as Galeterone and EPI-506 have been developed to effectively target and reduce AR-V7 levels as shown in cell line studies, with clinical trials underway [3]. Consequently, expression of AR-V7 together with that of full-length AR (AR-FL) have emerged as clinically relevant molecular biomarkers for CRPC [8,9]. AR-V7 is detectable in tissue at the RNA and protein levels, and can be evaluated using RNA hybridization techniques and immunohistology [10,11]. AR-V7 expression is rare in hormone-sensitive PC but correlates with CRPC [12]. However, in the advanced PC setting, tissue biopsies are generally unavailable for biomarker testing and diagnostic decision making. Liquid biopsies have in recent years emerged as an alternative tumor source for biomarker testing [13]. To date, AR-V7 has been detectable in circulating tumor cells (CTCs) isolated from blood samples, whole blood mRNA, from ctRNA, tumor arisen cellular vesicles, so-called exosomes found in plasma or serum and even urine [14,15,16,17,18,19]. Significantly, AR-V7 detection in CTCs has been associated with non-response to novel anti-androgens such as abiraterone and enzalutamide [14,20]. In contrast, the response rates to taxane-based chemotherapy showed no significant difference between AR-V7-positive versus-negative CRPC patients [14]. As such, this has positioned AR-V7 as a potential predictive biomarker that can discriminate between the use of an anti-androgen therapy and taxane chemotherapy.

**Figure 1:**
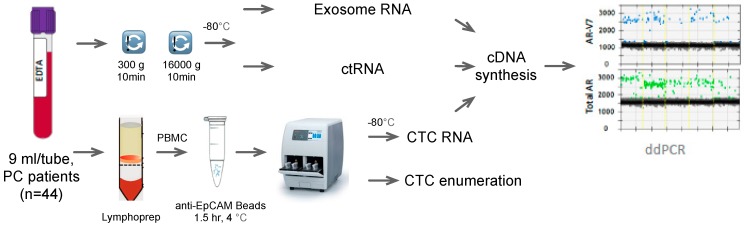
Work flow of the study. PC: prostate cancer, PBMC: peripheral blood mononuclear cells, ctRNA: circulating tumor ribonucleic acid, CTC: circulating tumor cell, cDNA: complementary deoxyribonucleic acid.

With AR-V7 emerging as a molecular biomarker of clinical importance, it is essential to determine the best strategy to detect this biomarker for predictive and prognostic purposes. Moreover, with blood biopsies emerging as a potential source to determine AR-V7 status, it is imperative to define which blood-based approach is the most sensitive and reliable. Herein we analyzed AR-V7 and AR-FL detectability in 44 PC patients using parallel blood samples for CTC isolation and ctRNA isolation. For 16 patients, parallel evaluation of AR-V7 and AR-FL status from exosomes was also possible (see Figure 1). Our data suggest that AR-V7 and AR-FL, while detectable from ctRNA and exosomal RNA, are most sensitively detected from CTC samples.

## 2. Materials and Methods 

### 2.1. Patients

The study was undertaken in accordance with the Declaration of Helsinki with human ethics approval, HREC/13/LPOOL/158, from the South Western Sydney Local Health District Ethics Committee (approval Sep 2013, extension July 2018). A total of 44 patients with prostate cancer were recruited at Liverpool Public and St George Private Hospitals in Australia. Patient information is summarized in Table 1. Per patient, 3 × 9 mL blood draws into 9 mL EDTA vacutubes (BD, Franklin Lakes, NJ, USA) were collected, 2 for CTC isolation and 1 for plasma preparation.

### 2.2. CTC Isolation

Peripheral blood mononuclear cells (PBMCs) including CTCs were isolated in parallel from two blood samples using Lymphoprep™ with SepMate™ tube-based gradient centrifugation (STEMCELL Technologies, Vancouver, BC, Canada) as per the manufacturer’s protocol. PBMCs were recovered and washed once with 5 mL phosphate-buffered saline (PBS) (Lonza, Basel, Switzerland), resuspended in 800 µL binding buffer with 40 µL FC buffer and 40 µL anti-EpCAM antibody coupled beads (IsoFlux CTC Enrichment kit, Fluxion Bioscience, San Francisco, CA, USA) and incubated for 90 min at 4 °C under gentle rotation. CTCs were then enriched using the standard IsoFlux CTC isolation protocol (Fluxion Bioscience, CA, USA). One CTC isolate underwent CTC enumeration and the other was immediately frozen at −80 °C until RNA extraction and gene expression analysis.

### 2.3. CTC Enumeration

IsoFlux enriched CTCs were enumerated with an IsoFlux CTC enumeration kit containing all the staining antibodies (Fluxion Bioscience, Alameda, CA, USA). Briefly, after 10 min fixation with fixation buffer, cells were blocked for 10 min with 10% normal donkey serum (NDS) and probed with rabbit anti-human CD45 antibody (1:100) followed by detection with tetramethylrhodamine (TRITC) conjugated donkey-anti rabbit IgG (1:200). Cells were then washed twice with binding buffer and permeabilized using 0.2% Trition 100 before probing with fluorescein (FITC) conjugated anti-human Pan-cytokeratin antibody (1:10). After two further washes, cells were transferred to SensoPlate™ 24-well glass bottom plates (Greiner Bio-one, Kremsmünster, Austria) with Hoechst dye included in the mounting media. Fluorescent microscopy (ALS CellCelector™, ALS, Jena, Germany) was used for CTC enumeration.

### 2.4. Plasma Processing

All plasma was processed within 4 h of blood draw. Whole blood was spun at 280× *g* for 10 min and the supernatant spun again using a microfuge at 16,000× *g* for 10 min at 4 °C. The supernatant was transferred and frozen at −80 °C until RNA extraction.

### 2.5. RNA Extraction and cDNA Synthesis

RNA from CTC samples was extracted with Total RNA Purification Micro Kit (Norgen Biotek, Thorold, ON, Canada) and eluted in 30 µL elution buffer. RNA from plasma was extracted with QIAamp circulating nucleic acid kit (QIAGEN, Hilden, Germany) and eluted in 65 µL elution buffer. Samples processed with this kit are referred to as ctRNA even for healthy controls that would only have normal cell free RNA. RNA from exosomes was extracted with Qiagen exoRNeasy serum/plasma Midi kit (QIAGEN, Hilden, Germany) and eluted in 30 µL. A total of 15 µL eluted RNA was reverse transcribed using SensiFAST cDNA synthesis kit (Bioline, Alexandria, Australia).

### 2.6. Droplet Digital PCR (ddPCR)

A total of 7 µL of cDNA of either CTC, ctRNA or exosomal samples was used for detection of AR-FL and AR-V7 transcripts by ddPCR as previously described [15]. Glyceraldehyde 3-phosphate dehydrogenase (GAPDH) transcript abundance was similarly evaluated (see Table 2 for primers, probes) and the optimized annealing temperature of 55 °C using the Bio-Rad QX200 ddPCR instrument (Bio-Rad, Hercules, CA, USA). Positive (cDNA from 22RV1 prostate cancer cells) and negative controls (DNase and RNase free H_2_O) were included in all ddPCR experiments.

### 2.7. Statistics

Data were analyzed with GraphPad Prism8 (GraphPad Software, San Diego, CA, USA). As a normal distribution was not assumed, non-parametric statistics was utilized. A Fisher exact test was used to test the significance between two categorical variables; *p* < 0.05 represents statistical significance. Unpaired comparison of CTC numbers between hormone sensitive prostate cancer (HSPC) and CRPC and AR copy numbers between healthy controls and patient samples was performed with the non-parametric Mann–Whitney test.

## 3. Results

### 3.1. Patients

Twelve HSPC and 32 CRPC patients at various stages of treatment were recruited, all patients had been originally diagnosed with subtype adenocarcinoma PC. Patient baseline characteristics are listed in Table 1. CTCs were enumerated and the expression levels of AR-FL and AR-V7 transcripts were detected in enriched CTC samples using our previously established ddPCR assay [15]. Extracted ctRNA and exosomal RNA were also tested by ddPCR for AR-FL and AR-V7 transcripts. To verify the quality of extracted RNA the reference gene GAPDH was tested for and detected in all samples (median GAPDH: 13212 copies/ml plasma, 2920-120606).

### 3.2. Prevalence of CTCs

**Figure 2:**
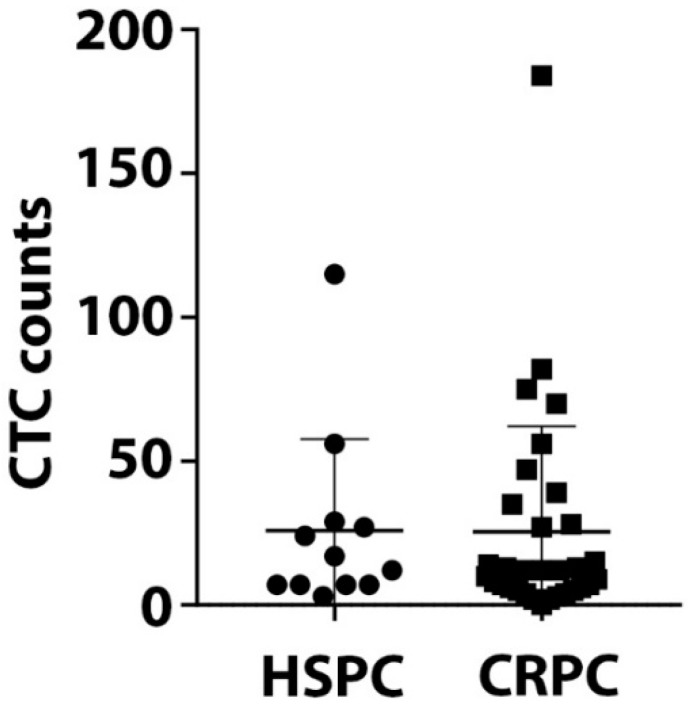
Circulating tumor cells (CTC) counts. CTCs were isolated using the IsoFlux CTC platform and enumerated. The range of CTC counts/9 mL blood for 12 HSPC and 32 CRPC patients is depicted. There is no significant difference of CTC numbers between HSPC and CRPC patients (*p* value = 0.59).

CTCs were detected in 97.7% (43/44) of patients. CTC counts varied from 0 to 184 per 9 mL blood with no significant difference in CTC counts between HSCP and CRPC patients (Figure 2).

### 3.3. AR-V7 and AR-FL in CTC RNA and Plasma ctRNA

**Figure 3:**
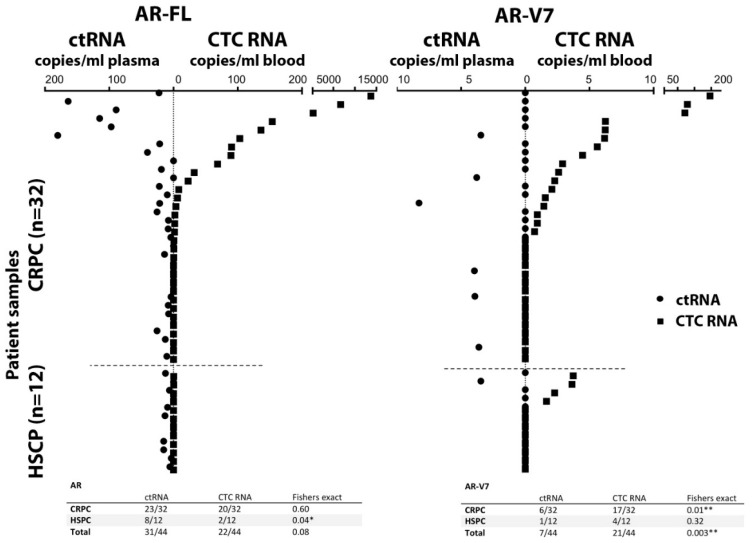
Detection of AR-FL (left) and AR-V7 (right) transcripts. Comparison of AR-FL and AR-V7 detection in CTC RNA and ctRNA from the same blood draw illustrated by mirrored scatter blot for the 44 patient samples (sorted in relation to detection in CTCs); summarized data are tabled below. *, ** indicate significance *p*-value ≤ 0.5 and ≤ 0.01, respectively.

As expected, AR-FL and AR-V7 was only found in CTC processed blood samples with detectable CTCs. AR-FL was detected in 22 out of 44 patient CTC samples (50%). AR-FL copy number detected for CTC samples ranged from 0 to 13,714 copies/mL blood. A 62.5% share (20/32) of CRPC samples had AR-FL positive CTC samples, and only 2 HSPC patients, 16.7%, had low copy numbers of AR-FL (Figure 3, Supplementary Table S1). For AR-V7, 47.7% (21/44) of patient CTC samples tested positive with 0–146 copies/ml blood. A share of 53.1% (17/32) of CRPC patients had detectable AR-V7 (range 0–146 copies/mL blood), while AR-V7 was also identified in 4 HSPC patient CTC samples, at generally lower levels (range 0–4 copies/mL blood).

Interestingly, AR-FL detection from plasma-derived ctRNA was higher, with 70.5% (31/44) patients testing positive, although detection frequency was not significantly different from CTC samples (Fisher exact, *p* = 0.13). The detected AR-FL copy numbers found in ctRNA ranged from 0 to 180 per ml plasma and were overall less abundant compared to CTC samples. Detection of AR-FL in ctRNA samples was similarly common in HSPC patients at 66.7% (8/12) compared to CRPC patients at 71.9% (23/32), although copy numbers tended to be higher in CRPC samples ranging between 0 and 15.2 vs. 0–180, for HSPC and CRPC patients, respectively.

In contrast, AR-V7 detection in ctRNA was lower in comparison to CTC samples with only 15.9% (7/44) of patient ctRNA samples testing positive. The difference in AR-V7 detectability in CTCs vs. ctRNA was significant (*p* = 0.003). AR-V7 tends to be more frequently found in CRPC patient ctRNA 18.8% (6/32) vs. 8.3% (1/12) in HSPC patient ctRNA, but detectability and copy numbers are generally low for both CRPC and HSPC patients (range 0–8 per mL plasma). This data indicates that both AR-FL and AR-V7 are more readily detectable in CTC samples.

In the 44-patient cohort, AR-FL detection in CTCs correlated with CRPC (*p* = 0.02) while AR-V7 detection did not reach a significant correlation with CRPC (*p* = 0.32). In contrast, no correlation was found for AR-FL in ctRNA (*p* = 0.73) or AR-V7 (*p* = 0.65) (Supplementary Table S1).

The concordance of AR-FL positivity between CTC and ctRNA was 41% (18/44) while concordance of AR-V7 positivity was only 9.1% (4/44), indicating lack of sensitivity and possibly specificity for AR-V7 detection in ctRNA.

### 3.4. AR-V7 and AR-FL in Exosomal RNA, ctRNA and CTCs

From 16 of the analyzed patient samples, enough plasma was available to additionally extract exosomal RNA and analyze for AR-FL and AR-V7 transcripts.

Overall, 12.5% (2/16) of patients were positive for AR-V7 when analyzed using exosomal RNA, compared to 6.3% (1/16) being considered AR-V7 positive according to ctRNA analysis in that patient sub-cohort. AR-V7 was also detectable in all CTC samples from the 16 patient cohort that were found to have detectable AR-V7 either by ctRNA or exosomal. Importantly however, overall more (43.8%, 7/16) patients tested positive for AR-V7 by CTC RNA analysis. In contrast, AR-FL was detected in 68.8% (11/16) ctRNA samples, in 50% (8/16) CTC samples and 37.5% (6/16) of exosomal RNA samples (Table 3).

### 3.5. Healthy Control Analysis and Implications for Assay Specificity

**Figure 4:**
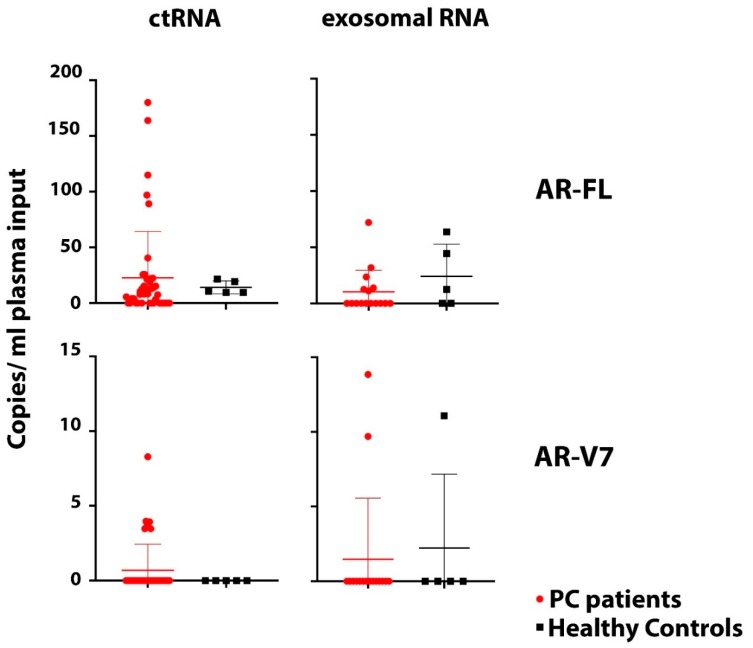
Comparison of AR-FL and AR-V7. AR-FL and AR-V7 detection in ctRNA (44 PC patients, 5 healthy controls) and exosomal RNA samples (16 PC patients and 5 healthy controls) indicates high background of AR transcript in plasma impacting specificity of tumor-derived AR-FL and AR-V7 detection. There is no significant difference of AR-FL and AR-V7 copy numbers detected in ctRNA and exosomal RNA between patients and healthy controls (*p* values: AR-FL ctRNA = 0.4; AR-FL exosomal = 0.32; AR-V7 ctRNA = 0.58; AR-V7 exosomal = 0.85).

We previously have demonstrated that AR-FL and AR-V7 detection from CTC samples is not only highly sensitive but also highly specific. When establishing the ddPCR assay we found AR-V7 undetectable even in large numbers of healthy donor PBMCs and AR-FL detection was very low (the equivalent of 0.001 or 0.002 copies per cell were detected in only 2 of 6 healthy donor PBMC samples; considering the average residual lymphocyte number in a CTC sample that would be equivalent to 0.44 or 0.88 copies per ml blood) [15]. To evaluate sensitivity and specificity of our AR assays when screening ctRNA and exosomal RNA samples, we obtained blood from five healthy individuals, age and sex matched to our patient cohort, and extracted ctRNA and exosomal RNA for AR-FL and AR-V7 analysis. AR-FL was readily detectable in ctRNA from all healthy subjects tested with an average copy number of 14.1 per ml plasma (range: 9.6–21.5). Of note, AR-FL copy numbers in only 34.1% (15/44) of our patient ctRNA samples were above that healthy control copy number average. AR-FL was also detected in exosomal RNA from three of five healthy subjects, and crucially, the copy number range was comparable to that detected in the exosomal patient samples. AR-V7 was not as prevalent but still present with 11.1 copies/mL plasma for one healthy individual when analyzing exosomal RNA while we detected very similar copy numbers, 13.9 and 9.7 copies per ml plasma, in the two exosomal RNA AR-V7 positive patients. AR-V7 was not detected in healthy donor ctRNA samples in this small healthy control cohort. Overall, these data strongly suggest the presence of variable and sometimes high levels of AR mRNA in healthy male plasma and exosomes, which clearly impacts reliable detection of PC-derived AR-FL/AR-V7 from ctRNA and exosomes and indicates low specificity for tumor-derived AR when testing ctRNA and exosomal RNA (Figure 4).

## 4. Discussion

Advancements in molecular technology have aided the accurate detection and quantification of novel blood-based biomarkers paving the way for their use in clinical environments. AR-V7 is emerging as a biomarker with the potential as a predictive tool in treatment selection. Patients that have detectable AR-V7 are considered to be resistant to abiraterone and enzalutamide but respond to taxane chemotherapy, and potentially eligible for clinical trials of new generation ADT drugs. Advanced stage PC tissue samples are generally unavailable for testing and liquid biopsies are explored as a surrogate. Thus far, studies on AR-V7 have utilized various methods of detection, resulting in different degrees of correlation between data and disease parameters, highlighting that a clear “gold standard” still needs to be found [21]. While previously developing a sensitive and specific AR-FL/AR-V7 detection method for enriched CTC samples [15], here we compared this method for detection of AR-FL and AR-V7 from CTC samples and ctRNA samples of 44 PC patients and for a subset of patient samples we also were able to compare detection from exosomal RNA. Exosomes in particular have become an attractive source of tumor information. They are small double lipid membrane vesicles of endocytic origin, that contain proteins, nucleic acids and lipids released by cells. Since this includes cancer cells, exosomes can be extracted from liquid biopsies such as plasma, serum and urine to be tested for biomarker information. Importantly, both ctRNA and exosomal RNA require simpler processing protocols than CTCs with commercially available kits and thus AR-FL/AR-V7 testing using these tumor information sources would potentially be easier translated into a clinical setting.

However, our data indicate CTC-based detection is superior in sensitivity and specificity for AR-V7 with a detection rate of 48% as compared to 16% with ctRNA. Nevertheless, both assays demonstrated some association of AR-V7 detection with patients classified as castrate resistant, although our cohort was too small to find significant correlation. Previous studies which utilize CTC enrichment-based techniques reported broad detection rates ranging from 27% to 75% [15,20,22,23]. These studies demonstrated a link between baseline CTC derived AR-V7 status and disease burden, which was found to increase with subsequent lines of therapy. Our data follows this trend, with the majority of our AR-V7 patients having had at least two lines of previous treatment (Supplementary Table S1). Our study, with a relatively small study patient cohort, was mainly aimed at comparing the effectiveness of choosing simple blood-based tumor sources rather than CTC samples to screen for AR-V7. We achieved this aim and our data indicates that CTC sample testing for PC derived AR-V7 and AR-FL is more sensitive and specific and thus ultimately more reliable.

Interestingly, the few patients detected positive for AR-V7 by either ctRNA or exosomal RNA testing were also found to be AR-V7 positive by CTC testing. Some CTC AR-V7 positive patients were however not detected as positive by ctRNA or exosomal RNA testing, suggesting higher sensitivity of the AR-V7 testing in CTC samples. We also detected AR-V7 in one healthy donor exosomal RNA sample. The implications of that for the individual remain unclear and further follow up is not possible because the individual consented as a healthy volunteer. Since the AR-V7 levels were not negligible and ddPCR data showed convincing detection, we have to assume poorer specificity of AR-V7 detection from exosomes at this stage, in comparison to that shown in our previous study for CTC samples [15]. However, larger healthy control studies would be necessary to confirm this.

Not all CTC samples tested positive for AR-FL when either parallel ctRNA or exosomal RNA testing detected it. However, this finding has to be interpreted together with the fact that we found quite high levels of AR-FL transcripts in ctRNA and exosomal RNA samples from age- and sex-matched healthy control individuals. This may not be surprising as exosomes and cell free nucleic acids are thought to be released during normal tissue homeostasis, and it is quite conceivable that AR-FL transcript is released into the blood stream in that way from organs like the testis and prostate in male subjects. By analyzing AR-FL from CTC samples we seem to largely avoid such transcripts from non-tumor sources as AR expression in normal blood cells is known to be minimal or null [24].

There may be other issues underpinning how, in comparison to ctRNA-based detection, CTC-derived AR-V7 provides for a more reliable assay. Given that high CTC counts on their own have proven to be of prognostic value in PC and detection of CTCs indicate higher disease burden, CTC detection can be interpreted hand-in-hand with AR detection to exclude potential false positives, and in our hands our ddPCR assay never detected AR-FL or AR-V7 in the absence of CTCs in a parallel enumerated sample [25]. ctRNA, on the other hand, due to the high levels of nucleases in the blood, has to be considered highly unstable and is also far more susceptible to variations in pre-analytical handling. Meanwhile, CTCs in blood appear to demonstrate superior stability and we have demonstrated previously that AR-V7 copies can be detected from CTC samples from patient blood drawn in simple EDTA tubes and stored up to 48 h at room temperature, suggesting CTCs either protect the AR-V7 transcript and/or continue to express it in a drawn blood sample [26].

Since exosomes in blood are similarly believed to protect the intravesicular content including mRNAs it was important to also test AR-V7 and AR-FL detection from exosomes. We detected AR-V7 less frequently than in parallel CTC samples in only two patients. While both patients with detectable AR-V7 from exosomes are classified as castrate resistant, we also detected similar AR-V7 copy numbers in one healthy subject, challenging reliability of the finding in PC patients.

While our small patient cohort was enough to determine the best liquid biopsy entity for AR-V7 and the study was not intended to answer questions of AR-V7 biology, there are a few issues worth highlighting. Firstly, our sensitive assay detected AR-V7 in some HSPC patient CTC samples. This is not entirely unexpected and has been reported previously [27,28]. It will be interesting to see whether AR-V7 detection in HSPC patients may be an early predictor of developing CRPC, as it tends to be associated with longer time on treatment in our study (AR-V7 positive HSPC patients: between 24 month and 60 month on ADT versus AR-V7 negative HSPC patients: being maximally treated for 12 month with ADT (see Supplementary Table S1)). Secondly, AR-V7 was found in some patient CTC samples despite undetectability of AR-FL. This has been reported by others [29] and it would be interesting to investigate the impacts of AR-V7 potentially totally replacing AR-FL in these patients in future studies. Finally, there seems to be a trend which matches previous reports [30] that patients on second line ADT have more commonly detectable AR-V7 in CTCs. In our study, 10 of 14 (69%) CRPC patients receiving enzalutamide and 5 of 9 (55.5%) receiving abiraterone (some patients were treated consecutively with both) were AR-V7 positive by CTC testing.

Although the small patient cohort allowed us to answer the main question of which liquid biopsy entity is the better for detection of AR-V7 and AR-FL, a limitation of the study is that correlation with disease parameters was not as informative as a bigger cohort would have been. Larger cohorts of healthy control comparisons would be able to better define background AR-FL and AR-V7 in plasma.

## 5. Conclusions

This study compared AR-V7 and AR-FL detection in liquid biopsy (blood)-derived CTC samples, ctRNA samples and exosomes. Our data show that testing of these clinically highly relevant biomarkers for PC patients is most reliable performed from CTC samples in regards to sensitivity and specificity. It also should be noted that a recent report shows that at the protein level correlation with PC disease parameters is linked to the nuclear localization of AR-V7 protein in CTCs [30]. We usually analyze two parallel blood samples, one for CTC enumeration, and we have recently amended this protocol to incorporate AR-V7 immunocytostaining and cellular localization screening. The second blood sample is screened for AR-V7 and AR-FL transcripts by ddPCR. Both tests go hand-in-hand to confirm AR-V7 presence and highlight the importance of analyzing CTCs rather than other circulating tumor entities. Our future studies will clarify whether both CTC tests cooperatively show correlation with resistance to ADT.

## Figures and Tables

**Table 1:** Patient Characteristics.

Patient	*n* = 44
Mean age (years)	77 (55–94)
Gleason score (%)	
≥7	34 (77%)
<7	4 (9%)
N.A.	6 (14%)
Metastatic status (%)	41 (93%)
Bone metastases	35 (79%)
Lymph metastases	22 (50%)
Visceral metastases	13 (29%)
Definitive therapy (%)	
Radical Prostatectomy	12 (27%)
Radiotherapy	12 (27%)
Both	2 (4%)
None	22 (50%)
Past systemic therapy (%)	
None	2 (4%)
ADT	42 (95%)
Chemotherapy	17 (38%)
Novel antiandrogens	
Enzalutamide	14 (31%)
Abiraterone	9 (20%)
Both	4 (9%)

N.A.: data not available, ADT: androgen deprivation therapy.

**Table 2:** GAPDH ddPCR primers and probe.

Name	Sequence
GAPDH-fwrd	CGGGAAGCTTGTCATCAATGG
GAPDH-rev	CTCCACGACGTACTCAGCG
GAPDH-probe	FAM-5′-TCTTCCAGGAGCGAGATCCCT-3-BQ1

**Table 3:** AR-FL/AR-V7 status in 16 patients tested from CTC, ctRNA and exosomal samples.

Patient	Status	AR-FL CTCs	AR-V7 CTCs	AR-FL ctRNA	AR-V7 ctRNA	AR-FL Exosomal	AR-V7 Exosomal
1	CRPC	−	−	−	−	+	−
2	CRPC	+	−	+	−	−	−
3	CRPC	+	−	+	−	−	−
4	CRPC	+	+	+	−	−	−
5	CRPC	−	+	+	−	+	+
6	CRPC	−	+	−	−	+	−
7	CRPC	+	+	+	−	+	+
8	CRPC	+	+	−	−	−	−
9	CRPC	+	−	+	−	−	−
10	HSPC	+	−	−	−	+	−
11	HSPC	+	−	+	−	−	−
12	HSPC	−	−	+	−	−	−
13	HSPC	−	+	+	−	−	−
14	HSPC	−	+	−	+	−	−
15	HSPC	−	−	+	−	+	−
16	HSPC	−	−	+	−	−	−

AR-V7 positive patients by CTC analysis are highlighted in grey.

## Supplementary Materials

Supplementary materials can be found at https://www.mdpi.com/2073-4409/8/7/688/s1.
