# Supplementary material for: Detection of AR-V7 in Liquid Biopsies of Castrate Resistant Prostate Cancer Patients: A Comparison of AR-V7 Analysis in Circulating Tumor Cells, Circulating Tumor RNA and Exosomes
Source: Cells. 2019 Jul 8;8(7):688. doi: 10.3390/cells8070688 (PMC6678978; doi:10.3390/cells8070688)
Supplement: Supplementary file 1 [file cells-08-00688-s001.pdf]

PATIENTS:

	Basic information			Metastsis				Primary treatment		Past systemic therapy		Noval therapy		CTC			ctRNA			Exosome		
Patient	Age	Hormone sensitivity status	Gleason score (High : ≥7 and Low: <7)	Metastatic disease	Bone metastases	Lymph metastases	Visceral metastases	Radical Prostatectomy	Radiation therapy	ADT (month on)	Chemo-therapy	Enzalutamide	Abiaterone	CTC count	AR-FL	AR-V7	GAPDH	AR-FL	AR-V7	GAPDH	AR-FL	AR-V7
1	65	HSPC	High	Yes	Yes	No	No	Yes	Yes	No	No	No	No	115	0	0	13212	6.23	0	2747	0	0
2	87	HSPC	High	Yes	Yes	No	No	No	No	Yes (6)	No	No	No	56	0	0	21818	15.24	0	-	-	-
3	80	HSPC	High	Yes	No	Yes	Yes	No	Yes	Yes (5)	No	No	No	29	0	0	11111	0	0	-	-	-
4	71	HSPC	N/A	Yes	Yes	No	Yes	No	No	Yes (N/A)	No	No	No	27	0.36	0	13576	12.47	0	1899	0	0
5	85	HSPC	High	Yes	No	No	Yes	No	No	Yes (6)	No	No	No	24	0.35	0	9790	0	0	3394	31.86	0
6	68	HSPC	High	No	No	No	No	No	Yes	Yes (24)	No	No	No	17	0	3.74	15272.73	13.16	0	824	0	0
7	86	HSPC	High	Yes	Yes	Yes	No	Yes	No	Yes (60)	No	No	No	12	0	3.64	9697	0	3.46	5939	0	0
8	62	HSPC	High	Yes	Yes	Yes	No	No	No	Yes (N/A)	Yes	No	No	7	0	1.66	28970	15.24	0	-	-	-
9	72	HSPC	High	No	No	No	No	Yes	No	No	No	No	No	7	0	0	14545	9.28	0	-	-	-
10	86	HSPC	N/A	Yes	Yes	Yes	No	No	No	Yes (12)	No	No	No	7	0	0	6751	3.46	0	2618	23.55	0
11	78	HSPC	High	Yes	Yes	No	No	No	No	Yes (6)	No	No	No	7	0	0	9256	5.67	0	2254	0	0
12	72	HSPC	High	Yes	Yes	No	No	Yes	No	Yes (24)	Yes	No	No	3	0	2.29	20606	0	0	-	-	-
13	80	CRPC	High	Yes	Yes	Yes	Yes	No	Yes	Yes	No	Yes	No	184	1.56	0	13731	8.31	0	-	-	-
14	65	CRPC	High	Yes	Yes	No	No	No	No	Yes	Yes	No	No	82	1.81	0	18874	7.92	3.96	-	-	-
15	71	CRPC	High	Yes	Yes	Yes	No	Yes	Yes	Yes	No	No	No	75	153.77	6.25	26886	114.95	0	-	-	-
16	84	CRPC	High	Yes	Yes	No	Yes	No	No	Yes	Yes	No	No	70	68.57	1.56	14269	0	0	994	0	0
17	90	CRPC	High	Yes	Yes	Yes	No	No	No	Yes	Yes	Yes	Yes	56	6701.3	146.49	28085	163.87	0	-	-	-
18	74	CRPC	High	Yes	Yes	Yes	No	No	Yes	Yes	Yes	Yes	No	47	89.35	0.73	21125	40.57	0	3442	72.03	9.7
19	94	CRPC	Low	Yes	Yes	No	No	No	No	Yes	No	No	No	39	319	2.3	N/A	89.16	3.78	-	-	-
20	77	CRPC	High	Yes	Yes	Yes	Yes	No	Yes	Yes	No	Yes	No	35	8.21	0	12764	22.05	0	4896.97	0	0
21	93	CRPC	High	Yes	Yes	Yes	No	No	No	Yes	No	No	No	28	32.21	0	18662	18.82	0	-	-	-
22	70	CRPC	High	Yes	No	Yes	Yes	Yes	No	Yes	Yes	Yes	No	27	90.39	71.69	29333	21.47	0	-	-	-
23	66	CRPC	N/A	Yes	Yes	Yes	No	Yes	No	Yes	Yes	Yes	No	15	6.23	6.23	16234	9.65	0	-	-	-
24	87	CRPC	High	Yes	Yes	No	Yes	No	No	Yes	No	No	No	14	0	0	5610	0	0	2109	12.47	0
25	88	CRPC	N/A	Yes	Yes	Yes	No	No	No	Yes	No	Yes	No	13	0.36	0	13175	13.99	0	-	-	-
26	82	CRPC	Low	Yes	Yes	No	No	Yes	No	Yes	No	No	No	13	0	0	7861	0	0	-	-	-
27	68	CRPC	High	Yes	No	Yes	No	No	No	Yes	No	No	No	12	13714.29	78.96	7273	22.46	0	-	-	-
28	70	CRPC	High	Yes	Yes	Yes	No	Yes	No	Yes	Yes	Yes	Yes	12	103.27	2.6	120606	180.09	0	5704.1	0	0
29	80	CRPC	High	Yes	Yes	Yes	Yes	No	No	Yes	Yes	Yes	No	12	136.1	6.18	13697	96.97	3.46	-	-	-
30	69	CRPC	N/A	Yes	Yes	Yes	Yes	No	No	Yes	No	No	Yes	10	0	0	2920	0	0	-	-	-
31	72	CRPC	N/A	Yes	Yes	Yes	Yes	No	No	Yes	Yes	Yes	No	9	0	2.91	15030	10.39	0	-	-	-
32	55	CRPC	Low	Yes	Yes	No	No	No	Yes	Yes	Yes	No	Yes	8	3.74	0	18424	21.47	0	4872.73	0	0
33	75	CRPC	High	Yes	Yes	Yes	Yes	No	No	Yes	Yes	Yes	No	7	0	1.45	13175	12.8	8.28	-	-	-
34	64	CRPC	High	Yes	No	No	Yes	Yes	No	Yes	No	No	No	7	0	0	6982	0	0	-	-	-
35	75	CRPC	High	Yes	Yes	No	No	No	No	Yes	Yes	Yes	Yes	6	22.86	0	16959	0	0	-	-	-
36	73	CRPC	High	No	No	No	No	No	No	Yes	No	No	No	6	0.73	0	8606	4.16	0	3182	0	0
37	94	CRPC	High	Yes	Yes	No	No	No	Yes	Yes	No	No	Yes	5	2.08	2.08	22909	25.63	0	-	-	-
38	72	CRPC	Low	Yes	Yes	No	No	No	Yes	Yes	Yes	Yes	No	5	0	4.47	7983	0	0	4169	11.08	0
39	65	CRPC	High	Yes	Yes	No	No	Yes	No	Yes	Yes	Yes	Yes	4	0	0.94	12242	25.63	0	1891	13.85	13.85
40	88	CRPC	High	Yes	Yes	No	No	No	No	Yes	No	No	No	4	0	0.94	11582	3.85	0	-	-	-
41	83	CRPC	High	Yes	Yes	Yes	No	No	Yes	Yes	No	No	No	3	0.57	0	13036	0	3.92	-	-	-
42	70	CRPC	High	Yes	No	Yes	No	Yes	No	Yes	No	No	No	2	0	0	7115	8.13	3.61	-	-	-
43	90	CRPC	High	Yes	Yes	Yes	No	No	No	Yes	No	No	Yes	2	0	5.61	6061	7.58	0	-	-	-
44	82	CRPC	High	Yes	Yes	No	No	No	Yes	Yes	Yes	No	Yes	0	0	0	6949	0	0	-	-	-

N/A: not available

AR-FL and AR-V7 for CTCs as copies/ml blood d AR-V7 for ctRNA and exosomes as copies/ml plasma

Healthy controls

	ctRNA		Exosomal RNA	
	AR-FL	AR-V7	AR-FL	AR-V7
HC1	21.47	0	63.72	0
HC2	19.39	0	44.33	11.08
HC3	9.6	0	0	0
HC4	10.67	0	12.47	0
HC5	9.6	0	0	0

AR or AR-V7 correlation with CRPC/HSPC status

CTC RNA

	AR-FL +	AR-FL -	AR-V7 +	AR-V7 -
**CRPC**	20	12	17	15
**HSPC**	2	10	4	8
**Fisher's exact test**		**p=0.02**		**p=0.32**

ct RNA

	AR-FL +	AR-FL -	AR-V7 +	AR-V7 -
**CRPC**	23	9	6	26
**HSPC**	8	4	1	11
**Fisher's exact test**		**p=0.73**		**p=0.65**
